# Small Sample Issues for Microarray-Based Classification

**DOI:** 10.1002/cfg.62

**Published:** 2001-02

**Authors:** Edward R. Dougherty

**Affiliations:** Department of Electrical Engineering Texas A&M University College Station TX 77843-3128 USA

## Abstract

In order to study the molecular biological differences between normal and diseased tissues, it is desirable to perform classification among diseases and stages of disease using microarray-based gene-expression values. Owing to the limited number of microarrays typically used in these studies, serious issues arise with respect to the design, performance and analysis of classifiers based on microarray data. This paper reviews some fundamental issues facing small-sample classification: classification rules, constrained classifiers, error estimation and feature selection. It discusses both unconstrained and constrained classifier design from sample data, and the contributions to classifier error from constrained optimization and lack of optimality owing to design from sample data. The difficulty with estimating classifier error when confined to small samples is addressed, particularly estimating the error from training data. The impact of small samples on the ability to include more than a few variables as classifier features is explained.


**Comparative and Functional Genomics**
*Comp Funct Genom* 2001; **2**: 28–34.

**Review Article**

### Small sample issues for microarray-based classification

Edward R. Dougherty*
*Department of Electrical Engineering, Texas A&M University, College Station, TX, USA*

*Correspondence to:
E. R. Dougherty, Department of Electrical Engineering, Texas A&M University, College Station, TX 77843-3128, USA.
E-mail: e-dougherty@tamu.edu*

### Abstract

**In order to study the molecular biological differences between normal and diseased tissues, it is desirable to perform classification among diseases and stages of disease using microarray-based gene-expression values. Owing to the limited number of microarrays typically used in these studies, serious issues arise with respect to the design, performance and analysis of classifiers based on microarray data. This paper reviews some fundamental issues facing small-sample classification: classification rules, constrained classifiers, error estimation and feature selection. It discusses both unconstrained and constrained classifier design from sample data, and the contributions to classifier error from constrained optimization and lack of optimality owing to design from sample data. The difficulty with estimating classifier error when confined to small samples is addressed, particularly estimating the error from training data. The impact of small samples on the ability to include more than a few variables as classifier features is explained. Copyright © 2001 John Wiley & Sons, Ltd.**

**Keywords:** classification; gene expression; genomics; microarrays; pattern recognition

### Introduction cDNA microarrays can provide expression measurements for thousands of genes at once [2,3,7]. A key goal is to perform classification via different expression patterns, e.g. cancer classification [4]. This requires designing a classifier (decision function) that takes a vector of gene expression levels as input, and outputs a class label, which predicts the class containing the input vector. Classification can be between different kinds of cancer, different stages of tumour development or a host of such differences. Classifiers are designed from a sample of expression vectors. This requires assessing expression levels from RNA obtained from the different tissues with microarrays, determining genes whose expression levels can be used as classifier variables, and then applying some rule to design the classifier from the sample microarray data. Expression values have randomness arising from both biological and experimental variability. Design, performance evaluation and application of classifiers must take this randomness into account.

Three critical issues arise. First, given a set of variables, how does one design a classifier from the sample data that provides good classification over the general population? Second, how does one estimate the error of a designed classifier when data is limited? Third, given a large set of potential variables, such as the large number of expression level determinations provided by microarrays, how does one select a set of variables as the input vector to the classifier? The problem of error estimation impacts variable selection in a devilish way. An error estimator may be unbiased but have a large variance and therefore often be low. This can produce a large number of gene (variable) sets and classifiers with low error estimates. For a small sample, we can end up with thousands of gene sets for which the error estimate from the data at hand is zero.

For at least the near future, small samples are likely to be a critical issue for microarray-based classification. The irony is that, while microarray technology yields information on very large gene sets, it is just these large sets that demand experimental replication. If detectors for each gene are not duplicated on an array, then one microarray

Copyright © 2001 John Wiley & Sons, Ltd.

yields a single sample point per gene. In this case, a study using 30 arrays provides a very small sampling of gene behaviour. This paper discusses classification issues, with particular attention to the perplexing effect of small samples.

### Classification rules

Classification involves a *classifier*, $\psi$, a *feature vector*, $\mathbf{X}=(X_1, X_2,\ldots, X_d)$ composed of random variables, and a binary random variable, $Y$, to be predicted by $\psi(\mathbf{X})$. The values, 0 or 1, of $Y$ are treated as class labels. The error, $\varepsilon(\psi)$, of $\psi$ is the probability, $P(\psi(\mathbf{X})\neq Y)$, that the classification is erroneous. It equals the expected (mean) absolute difference, $E(|Y-\psi(\mathbf{X})|)$, between the label and the classification. $X_1, X_2,\ldots, X_d$ can be discrete or real-valued. In the latter case, the domain of $\psi$ is $d$-dimensional Euclidean space $\Re^d$. An optimal classifier, $\psi_\bullet$, is one having minimal error, $\varepsilon_\bullet$, among all binary functions on $\Re^d$. $\psi_\bullet$ and $\varepsilon_\bullet$ are called the *Bayes classifier* and *Bayes error*, respectively. Classification accuracy, and thus the error, depends on the probability distribution of the feature–label pair ($\mathbf{X}$, $Y$)—how well the labels are distributed among the variables (gene expression levels) being used to discriminate them, and how the variables are distributed in $\Re^d$.

The Bayes classifier is defined in a natural way: for any specific vector $\mathbf{x}$, $\psi_\bullet(\mathbf{x})=1$ if the expected value of $Y$ given $\mathbf{x}$, $E(Y|\mathbf{x})$, exceeds ½, and $\psi_\bullet(\mathbf{x})=0$ otherwise. Formulated in terms of probabilities, $\psi_\bullet(\mathbf{x})=1$ if the conditional probability of $Y=1$ given $\mathbf{x}$ exceeds the conditional probability of $Y=0$ given $\mathbf{x}$, and $\psi_\bullet(\mathbf{x})=0$ otherwise; that is, $\psi_\bullet(\mathbf{x})=1$ if and only if $P(Y=1|\mathbf{x})>P(Y=0|\mathbf{x})$. This is most intuitive: the label 1 is predicted upon observation of $\mathbf{x}$ if the probability that $\mathbf{x}$ lies in class 1 exceeds the probability that $\mathbf{x}$ lies in class 0. Since the sum of the probabilities is 1, $P(Y=1|\mathbf{x})>P(Y=0|\mathbf{x})$ if and only if $P(Y=1|\mathbf{x})>½$. The problem is that we do not know these conditional probabilities, and therefore must design a classifier from sample data.

Supervised classifier design uses a *sample* $S_n=[(\mathbf{X}^1, Y^1), (\mathbf{X}^2, Y^2),\ldots, (\mathbf{X}^n, Y^n)]$ of feature–label pairs and a *classification rule* to construct a classifier $\psi_n$ whose error is hopefully close to the Bayes error. The Bayes error $\varepsilon_\bullet$ is estimated by the error $\varepsilon_n$ of $\psi_n$. Because $\varepsilon_\bullet$ is minimal, $\varepsilon_n\geq\varepsilon_\bullet$, and there is a design error (cost of estimation), $\Delta_n=\varepsilon_n-\varepsilon_\bullet$. Since it depends on the sample, $\varepsilon_n$ is a random variable, as is $\Delta_n$. Hopefully, $\Delta_n$ gets closer to 0 as the sample size grows. This will depend on the classification rule and the distribution of the feature–label pair ($\mathbf{X}$, $Y$).

A classification rule is said to be *consistent* for the distribution of ($\mathbf{X}$, $Y$) if $E(\Delta_n)\to 0$ as $n\to\infty$, where the expectation is relative to the distribution of the sample. The expected design error goes to zero as the sample size goes to infinity. This is equivalent to $P(\Delta_n>\tau)\to 0$ as $n\to\infty$ for any $\tau>0$, which says that the probability of the design error exceeding $\tau$ goes to 0. As stated, consistency depends upon the relation between the classification rule and the joint feature–label distribution. If $E(\Delta_n)\to 0$ for any distribution, then the classification rule is said to be *universally consistent*. Since we often lack an estimate of the distribution, universal consistency is desirable.

Since the Bayes classifier is defined by $\psi_\bullet(\mathbf{x})=1$ if and only if $P(Y=1|\mathbf{x})>½$, an obvious way to proceed is too obtain an estimate $P_n(Y=1|\mathbf{x})$ of $P(Y=1|\mathbf{x})$ from the sample $S_n$. The *plug-in rule* designs a classifier by $\psi_n(\mathbf{x})=1$ if and only if $P_n(Y=1|\mathbf{x})>½$. If the data is discrete, then there is a finite number of vectors and $P_n(Y=1|\mathbf{x})$ can be defined to be the number of times the pair ($\mathbf{x}$, 1) is observed in the sample divided by the number of times $\mathbf{x}$ is observed. The problem is that, if $\mathbf{x}$ is observed very few times, then $P_n(Y=1|\mathbf{x})$ is not a good estimate. Even worse, if $\mathbf{x}$ is never observed, then $\psi_n(\mathbf{x})$ must be defined by some convention. The rule is consistent, but depending on the number of variables, may require a large sample to have $E(\Delta_n)$ close to 0, or equivalently, $\varepsilon_n$ close to the Bayes error. Consistency is of little consequence for small samples.

For continuous data, many classification rules partition $\Re^d$ into a disjoint union of cells. $P_n(Y=1|\mathbf{x})$ is the number of 1-labelled sample points in the cell containing $\mathbf{x}$ divided by the total number of points in the cell. A *histogram rule* is defined by the plug-in rule: $\psi_n(\mathbf{x})$ is 0 or 1 according to which is the majority label in the cell. The cells may change with $n$ and may depend on the sample points. They do not depend on the labels. To obtain consistency for a distribution, two conditions are sufficient when stated with the appropriate mathematical rigour: (1) the partition should be fine enough to take into account local structure of the

Copyright © 2001 John Wiley & Sons, Ltd.

distribution, and (2) there should be enough labels in each cell so that the majority decision reflects the decision based on the true conditional probabilities.

The cubic histogram rule partitions $\Re^d$ into same-size cubes. These can remain the same or vary with sample size $n$. If the cube edge length approaches 0 and $n$ times the common volume approaches infinity as $n \to \infty$, then the rule is universally consistent. For discrete data, the cubic histogram rule reduces to the plug-in rule for discrete data if the cubes are sufficiently small.

Another popular rule is the *nearest-neighbour* (NN) *rule*. $\psi_n(\mathbf{x})$ is the label of the sample point closest to $\mathbf{x}$. This rule is simple, but not consistent. An extension of this rule is the *k-nearest-neighbour* (*k*NN) *rule*. For $k$ odd, the $k$ points closest to $\mathbf{x}$ are selected and $\psi_n(\mathbf{x})$ is defined to be 0 or 1 according to which is the majority among the labels of the chosen points. The *k*NN is universally consistent if $k \to \infty$ in such a way that $k/n \to 0$ as $n \to \infty$.

### Constrained Classifiers

To reduce design error, one can restrict the functions from which an optimal classifier must be chosen to a class C. This leads to trying to find an optimal *constrained* classifier, $\psi_C \in C$, having error $\varepsilon_C$. Constraining the classifier can reduce the expected design error, but at the cost of increasing the error of the best possible classifier. Since optimization in C is over a subclass of classifiers, the error, $\varepsilon_C$, of $\psi_C$ will typically exceed the Bayes error, unless the Bayes classifier happens to be in C. This *cost of constraint* (*approximation*) is $\Delta_C = \varepsilon_C - \varepsilon_\bullet$. A classification rule yields a classifier $\psi_{n,C} \in C$ with error $\varepsilon_{n,C}$, and $\varepsilon_{n,C} \geq \varepsilon_C \geq \varepsilon_\bullet$. Design error for constrained classification is $\Delta_{n,C} = \varepsilon_{n,C} - \varepsilon_C$. For small samples, this can be substantially less than $\Delta_n$, depending on C and the rule. The error of the designed constrained classifier is decomposed as $\varepsilon_{n,C} = \varepsilon_\bullet + \Delta_C + \Delta_{n,C}$. The expected error of the designed classifier from C can be decomposed as:

$$E(\varepsilon_{n,C}) = \varepsilon_\bullet + \Delta_C + E(\Delta_{n,C}) \qquad (1)$$

The constraint is beneficial if and only if $E(\varepsilon_{n,C}) < E(\varepsilon_n)$, which means $\Delta_C < E(\Delta_n) - E(\Delta_{n,C})$. If the cost of constraint is less than the decrease in expected design cost, then the expected error of $\psi_{n,C}$ is less than that of $\psi_n$. The dilemma: strong constraint reduces $E(\Delta_{n,C})$ at the cost of increasing $\varepsilon_C$.The matter can be graphically illustrated. For the discrete-data plug-in rule and the cubic histogram rule with fixed cube size, $E(\Delta_n)$ is non-increasing, meaning that $E(\Delta_{n+1}) \leq E(\Delta_n)$. This means that the expected design error never increases as sample sizes increase, and it holds for any feature–label distribution. Such classification rules are called '*smart*'. They fit our intuition about increasing sample sizes. The nearest-neighbour rule is not smart because there exist distributions for which $E(\Delta_{n+1}) \leq E(\Delta_n)$ does not hold for all $n$. Now consider a consistent rule, constraint, and distribution for which $E(\Delta_{n+1}) \leq E(\Delta_n)$ and $E(\Delta_{n+1,C}) \leq E(\Delta_{n,C})$. Then Figure 1 illustrates the design problem. The axes correspond to sample size and error. The horizontal dashed and solid lines represent $\varepsilon_\bullet$ and $\varepsilon_C$, respectively; the decreasing dashed and solid lines represent $E(\varepsilon_n)$ and $E(\varepsilon_{n,C})$, respectively. If $n$ is sufficiently large, then $E(\varepsilon_n) < E(\varepsilon_{n,C})$; however, if $n$ is sufficiently small, then $E(\varepsilon_n) > E(\varepsilon_{n,C})$. The point $N_0$ at which the decreasing lines cross is the cut-off: for $n > N_0$, the constraint is detrimental; for $n < N_0$, it is beneficial. When $n < N_0$, the advantage of the constraint is the difference between the decreasing solid and dashed lines.

There are many kinds of constrained classifiers. *Perceptrons* form a constrained class with some attractive properties: simplicity, a linear-like structure, and contributions of individual variables that can be easily appreciated. Savings in sample size (in comparison to unconstrained classification) accelerate as the number of variables increases.

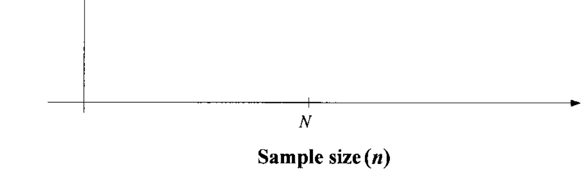

**Figure 1.** Relation between sample size and constraint

Copyright © 2001 John Wiley & Sons, Ltd.

A perceptron is defined by:

$$\psi(\mathrm{X}) = \mathrm{T}(a_1X_1 + a_2X_2 + \cdots + a_mX_m + b) \qquad (2)$$

where T is a threshold function, $\mathrm{T}(z)=0$ if $z \leq 0$, and $\mathrm{T}(z)=1$ if $z>0$. A perceptron splits $\Re^d$ into two by the hyperplane defined by setting the sum in the preceding equation to 0. Design of a perceptron requires estimating the coefficients $a_1, a_2, \ldots, a_m$, and $b$.

*Neural networks* are multi-layer perceptrons. A basic two-layer neural network takes the outputs of $K$ perceptrons (called *neurons*) and inputs these outputs into a final perceptron. More general networks exist. Neural networks offer an advantage over perceptrons because by increasing the number of neurons one can arbitrarily decrease the constraint. But this makes neural networks tricky to use because decreasing the constraint increases the expected design cost. One faces the inevitable conundrums of balancing the contributions to $E(\varepsilon_{n,\mathrm{C}})$ in Eq. 1. The data requirement grows rapidly as the number of neurons is increased.

### Error Estimation

The error of a designed classifier needs to be estimated. If there is an abundance of data, then it can be split into *training* and *test* data. A classifier is designed on the training data. Its estimated error is the proportion of errors it makes on the test data. The estimate is unbiased and its variance tends to zero as the amount of test data goes to infinity.

A problem arises when data are limited. One approach is to use all sample data to design a classifier $\psi_n$, and estimate $\varepsilon_n$ by applying $\psi_n$ to the same data. The *resubstitution estimate*, $\underline{\varepsilon}_n$, is the fraction of errors made by $\psi_n$. For histogram rules, $\underline{\varepsilon}_n$ is biased low, meaning $E(\underline{\varepsilon}_n) \leq E(\varepsilon_n)$. For small samples, the bias can be severe. It improves for large samples. For binary features, an upper bound for the mean-square error of $\underline{\varepsilon}_n$ as an estimator of $\varepsilon_n$ is given by $E(|\underline{\varepsilon}_n - \varepsilon_n|^2) \leq 6(2^d)/n$. Note the exponential contribution of the number of variables. Figure 2 shows a generic situation for the inequality $E(\underline{\varepsilon}_n) \leq E(\varepsilon_\bullet) \leq E(\varepsilon_n)$ for increasing sample size.

To appreciate the problem with resubstitution, consider the plug-in rule for discrete data. For any vector $\mathbf{x}$, let $n(\mathbf{x})$ be the number of occurrences of $\mathbf{x}$ in the sample data, $n(Y=1|\mathbf{x})$ be the number of times $\mathbf{x}$ has label 1, and $P_n(Y=1|\mathbf{x}) = n(Y=1|\mathbf{x})/n(\mathbf{x})$.

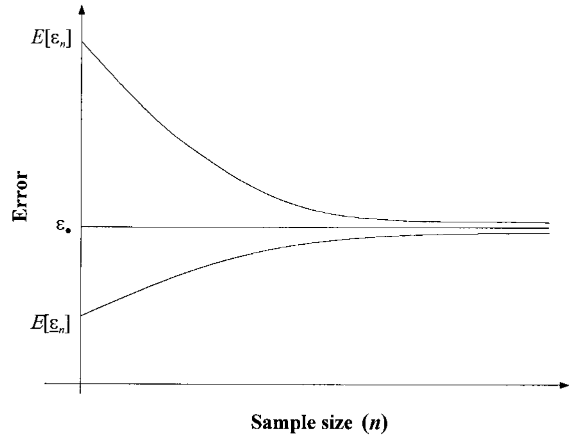

**Figure 2.** Expected design error vs. expected resubstitution error

There are three possibilities: (1) $\mathbf{x}$ is observed in training, $n(Y=1|\mathbf{x}) > n(\mathbf{x})/2$, $P_n(Y=1|\mathbf{x}) > ½$, and $\psi_n(\mathbf{x})=1$; (2) $\mathbf{x}$ is observed in training, $n(Y=1|\mathbf{x}) \leq n(\mathbf{x})/2$, $P_n(Y=1|\mathbf{x}) \leq ½$, and $\psi_n(\mathbf{x})=0$; or (3) $\mathbf{x}$ is not observed in training and $\psi_n(\mathbf{x})$ is defined by a convention. Each $\mathbf{x}$ in the first category contributes $n(Y=0|\mathbf{x})$ errors. Each $\mathbf{x}$ in the second category contributes $n(Y=1|\mathbf{x})$ errors. For a small sample, there may be an enormous number of vectors in the third category. These contribute nothing to $\underline{\varepsilon}_n$, but may contribute substantially to $\varepsilon_n$. Moreover, there may be many vectors in the first and second categories observed only once, and they also contribute nothing to $\underline{\varepsilon}_n$.

Another small-sample approach is *cross-validation*. Classifiers are designed from parts of the sample, each is tested on the remaining data, and $\varepsilon_n$ is estimated by averaging the errors. For *leave-one-out estimation*, $n$ classifiers are designed from sample subsets formed by leaving out one sample pair. Each is applied to the left-out pair, and the estimator $\hat{\varepsilon}_n$ is $1/n$ times the number of errors made by the $n$ classifiers. Since the classifiers are designed on sample sizes of $n-1$, $\hat{\varepsilon}_n$ actually estimates the error $\varepsilon_{n-1}$. It is an unbiased estimator of $\varepsilon_{n-1}$, meaning that $E(\hat{\varepsilon}_n) = E(\varepsilon_{n-1})$. Unbiasedness is important, but of critical concern is the variance of the estimator for small $n$.

For a sample of size $n$, $\hat{\varepsilon}_n$ estimates $\varepsilon_n$ based on the same sample. Performance depends on the classification rule. For the $k$-nearest-neighbour rule, $E(|\hat{\varepsilon}_n - \varepsilon_n|^2) \leq (6k+1)/n$. Given that $\hat{\varepsilon}_n$is approximately an unbiased estimator of $\varepsilon_n$, this

Copyright © 2001 John Wiley & Sons, Ltd.

inequality bounds the variance of $\hat{\varepsilon}_n - \varepsilon_n$. Although an upper bound does not say how bad the situation is, but only how bad it can at most be, it can be instructive to look at its order of magnitude. For $k=1$ and $n=175$, upon taking the square root, this bound only ensures that the standard deviation of $\hat{\varepsilon}_n - \varepsilon_n$ is less than 0.2.

It is informative to compare the resubstitution and leave-one-out estimates for the histogram rule. The variance of the resubstitution estimator is bounded above by $1/n$, and if the partition on which it is based contains $N$ cells, then $E(|\underline{\varepsilon}_n - \varepsilon_n|^2) \leq 6N/n$. For the leave-one-out estimator:

$$E[|\hat{\varepsilon}_n - \varepsilon_n|^2] \leq \frac{1+6e^{-1}}{n} + \frac{6}{\sqrt{\pi(n-1)}} \quad (3)$$

[see (1) for bounds]. $\sqrt{n-1}$ as opposed to $n$ in the denominator for $\underline{\varepsilon}_n$ shows greater variance for $\hat{\varepsilon}_n$. There is a certain tightness to this bound. For any partition there is a distribution for which:

$$E[|\hat{\varepsilon}_n - \varepsilon_n|^2] \geq \frac{1}{e^{1/12}\sqrt{2\pi n}} \quad (4)$$

Performance can be very bad for small $n$. Unbiasedness comes with increased variance.

To appreciate the difficulties inherent in the leave-one-out bounds, we will simplify them in a way that makes them more favourable to precise estimation. The performance of $\hat{\varepsilon}_n$ guaranteed by Eq. 3 becomes better if we lower the bound. A lower bound than the one in Eq. 3 is $(1.8)/\sqrt{n-1}$. The corresponding standard-deviation bounds for $n=50$ and 100 exceed 0.5 and 0.435, respectively. These are essentially useless. The minimum worst-case-performance bound of Eq. 4 would be better if it were lower. A lower bound than the one given is $(0.35)/\sqrt{n}$. The corresponding standard-deviation bounds for $n=50$ and 100, exceed 0.22 and 0.18, respectively.

Returning to the situation in which the data is split into *training* and *test* data, if the test-data error estimate is $\bar{\varepsilon}_n$ and there are $m$ sample pairs in the test data, then $E[|\bar{\varepsilon}_n - \varepsilon_n|^2] \leq 1/4m$. The problem is that, for small samples, one would like to use all the data for design. It is necessary to use 25 sample pairs for test data to get the corresponding standard-deviation bound down to 0.1.

### Feature Selection

Given a large set of potential features, such as the set of all genes on a microarray, it is necessary to find a small subset with which to classify. There are various methods of choosing feature sets, each having advantages and disadvantages. The typical intent is to choose a set of variables that provide good classification. The basic idea is to choose variables that are not redundant.

A critical problem arises with small samples. Given a large set of variables, every subset is a potential feature set. For $v$ variables, there are $2^v - 1$ possible feature vectors. Even for choosing from among 200 variables and allowing at most 20 variables, the number of possible vectors is astronomical. One cannot apply a classification rule to all of these; nonetheless, even if the classes are moderately separated, one may find many thousands of vectors for which $\hat{\varepsilon}_n \approx 0$. It would be wrong to conclude that the Bayes errors of all the corresponding classifiers are small.

Adjoining variables stepwise to the feature vector decreases the Bayes error but can increase design error. For fixed sample size $n$ and different numbers of variables $d$, Figure 3 shows a generic situation for the Bayes error $\varepsilon_\bullet(d)$ and the expected error $E[\varepsilon_n(d)]$ of the designed classifier as functions of $d$. $\varepsilon_\bullet(d)$ decreases; $E[\varepsilon_n(d)]$ decreases and then increases. Were $E[\varepsilon_n(d)]$ known, then we could conclude that $\varepsilon_\bullet(d)$ is no worse than $E[\varepsilon_n(d)]$; however, we have only an estimate of $\varepsilon_n(d)$, which for small samples can be well below (or above) $\varepsilon_\bullet(d)$. Thus, the estimate curve $\hat{\varepsilon}_n(d)$ might drop far

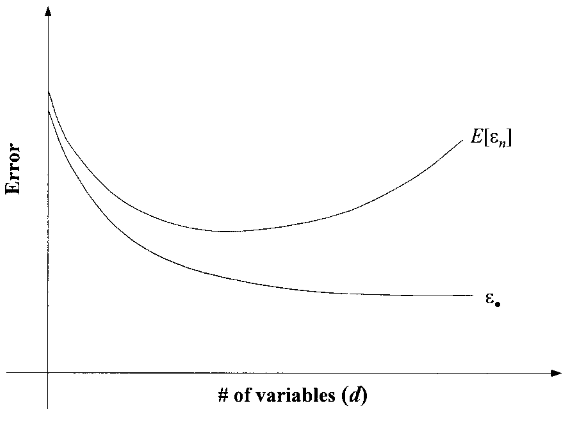

**Figure 3.** Effect of increasing numbers of variables

Copyright © 2001 John Wiley & Sons, Ltd.

below the Bayes-error curve $\varepsilon_{\bullet}(d)$, even being 0 over a fairly long interval.

We confront the general issue of the number of variables. The expected design error is written in terms of $n$ and $C$ in Eq. 1. But $C$ depends on $d$. A celebrated theorem of pattern recognition provides bounds for $E(\Delta_{n,C})$ [8]. The *empirical-error rule* chooses the classifier in $C$ that makes the least number of errors on the sample data. For this (intuitive) rule, $E(\Delta_{n,C})$ satisfies the bound:

$$E(\Delta_{n,C}) \leq 4\sqrt{\frac{V_C \log n + 4}{2n}} \tag{5}$$

where $V_C$ is the *VC* (*Vapnik–Chervonenkis*) *dimension* of $C$. Details of the VC dimension are outside the scope of this paper. Nonetheless, it is clear from Eq. 5 that $n$ must greatly exceed $V_C$ for the bound to be small. The VC dimension of a perceptron is $d+1$. For a neural network with an even number, $k$, of neurons, the VC dimension has the lower bound $V_C \geq dk$. If $k$ is odd, then $V_C \geq d(k-1)$. To appreciate the implications, suppose $d=k=10$. Setting $V_C=100$ and $n=5000$ in Eq. 5 yields a bound exceeding 1, which says nothing. Admittedly, the bound of Eq. 5 is worst-case because there are no distributional assumptions. The situation may not be nearly so bad. Still, one must proceed with care, especially in the absence of distributional knowledge. Adding variables and neurons is often counterproductive unless there is a large sample available. Otherwise, one could end up with a very bad classifier whose error estimate is very small!

### Conclusion

The purpose of this review has been to provide the general micorarray community with some basic guideposts in its effort to design expression-based classifiers. There are many more implications of the kind discussed here. In some sense, we have been discussing a worst-case setting: no assumptions on the distribution of features and labels, and real-valued variables. The data requirement can be significantly reduced if some prior knowledge concerning the distribution is applied, or if a strong constraint based on biological knowledge is imposed. The data problem can also be mitigated if the classifier variables are discrete and limited in their possible values. Two possibilities naturally arise. The Boolean model has been suggested for genomic networks, and could be used here instead of considering raw expression values [5]. In it, a gene is either *on* (1) or *off* (0). Ternary values are also appropriate for microrarray ratio data: a gene is *upregulated* (1), *downregulated* $(-1)$, or *invariant* (0). This model has been used to measure gene interaction via expression ratios [6]. One might reasonably argue that compression of the continuous data gives up too much information; however, given the data variability, it might be safer only to consider genes that change significantly, and base classification on an up–down model of control.

Most likely, it will not be possible to design a classifier from a single set of microarray experiments. Separation of the sample data by designed classifiers will likely have to be taken as evidence that the corresponding gene sets are potential variable sets for classification. Their effectiveness will have to be checked by large-replicate experiments designed to estimate their classification error, perhaps in conjunction with biological input or phenotype evidence. There may, in fact, be many gene sets that provide accurate classification of a given pathology. Of these, some sets may provide mechanistic insights into the molecular aetiology of the disease, while other sets may be indecipherable. This listing of difficulties in producing accurate classifiers based on measurements of the expression profiles of small samples is not intended to persuade researchers to cease doing experiments and subsequent analysis to arrive at indications that certain conditions can be discriminated via gene expression. Rather, it is intended to focus attention on the need to find classification screening algorithms that provide reasonable collections of gene sets to be tested with new experiments.

### References

1. Devroye L, Gyorfi L, Lugosi G. 1996. *A Probabilistic Theory of Pattern Recognition*. Springer-Verlag: New York.
2. De Risi JL, Iyer VR, Brown PO. 1997. Exploring the metabolic and genetic control of gene expression on a genomic scale. *Science* **278**: 680–666.
3. Duggan DJ, Bittner ML, Chen Y, Meltzer PS, Trent JM. 1999. Expression profiling using cDNA microarrays. *Nature Genet* **21**: 10–14.
4. Golub TR, Slonim DK, Tamayo P, *et al.* 1999. Molecular classification of cancer: class discovery and class prediction by gene expression monitoring. *Science* **286**: 531–537.

Copyright © 2001 John Wiley & Sons, Ltd.

5. Kauffman SA. 1993. *The Origins of Order*. Oxford University Press: New York.
6. Kim S, Dougherty ER, Bittner M, *et al*. 2000. General framework for the analysis of multivariate gene interaction via expression arrays. *Biomed Opt* **5**: 411–424.
7. Schena M, Shalon D, Davis RW, Brown PO. 1995. Quantitative monitoring of gene expression patterns with a complementary DNA microarray. *Science* **270**: 467–470.
8. Vapnik V, Chervonenkis A. 1971. On the uniform convergence of relative frequencies of events to their probabilities. *Theor Prob Appl* **16**: 264–280.

*The*
**Genomics**
*Website at Wiley*

**www.wiley.co.uk/genomics**

The **Genomics** website at Wiley is a **DYNAMIC** resource for the genomics community, offering **FREE** special feature articles and new information **EACH MONTH**.

Find out more about *Comparative and Functional Genomics*, and *Proteomics*, and how to view many articles **FREE OF CHARGE**!

Visit the **Library** for hot books in Genomics, Bioinformatics, Molecular Genetics and more.

Click on **Journals** for information on all our up-to-the minute journals, including: *Genesis, Bioessays, Gene Function and Disease, Human Mutation, Genes, Chromosomes and Cancer* and the *Journal of Gene Medicine*.

Let the **Genomics** website at Wiley be your **guide** to genomics-related web sites, manufacturers and suppliers, and a calendar of conferences.

WILEY

2171

Copyright © 2001 John Wiley & Sons, Ltd. 
